# Retraction: Placental expression of CD100, CD72 and CD45 is dysregulated in human miscarriage

**DOI:** 10.1371/journal.pone.0225491

**Published:** 2019-11-14

**Authors:** The PLOS ONE Editors

After this article [1] was published, the following concerns were raised about the results reported in Figs 5, 7 and 9:

1. Fig 5: There appear to be vertical discontinuities after lanes 2, 4 of the first trimester nCD100 panel, after lane 3 of the first MC nCD100 panel, after lanes 2, 3, 4, 5 of the second MC nCD100 panel.
2. Fig 7: There are similarities between lanes 1 and 2 in the second MC CD72 panel.
3. Fig 7: There are similarities between lanes 1 and 2 in the third MC CD72 panel.
4. It appears as though the same β-actin panels were used for all corresponding panels in Figs 5, 7 and 9.
5. Fig 7: There appears to be a vertical discontinuity after lane 3 of the third MC CD72 blot.
6. Fig 9: There appear to be vertical discontinuities after lanes 2 and 4 in the first trimester CD45 blot, and after lanes 2, 3 of the second MC CD45 blot.
7. Fig 9, first trimester CD45 blot: There are similarities in background signal above and below the bands in lanes 1, 3, and there are similarities in background signal below the bands in lanes 2, 4.
8. Fig 9, second MC CD45 blot: There are similarities in background signal above the bands in lanes 1, 2, and there are similarities in background signal above the bands in lanes 5, 6.
9. Fig 9 fourth MC CD45 blot: There are regions of similarity in background signal/pixilation below the bands in lanes 2, 3, 4, 5, when lane 5 is flipped horizontally. There are also similarities between background below the bands in lanes 1 and 6, when one is flipped horizontally.
10. Fig 9: There are similarities between lane 2 of the first MC CD45 blot and lane 2 of the second MC CD45 blot.
11. Within Figs 5, 7 and 9, the β-actin panels appear similar for the first and third MC panels.
12. Within Figs 5, 7 and 9, the β-actin panels appear similar for the first trimester and second MC.
13. Within Figs 5, 7 and 9, there are similarities between lanes 1–4 of the β-actin panels for first trimester and fourth MC.
14. Within Figs 5, 7 and 9, there are similarities between the Tonsil β-actin data and the data in lane 2 of the Term placenta β-actin blot.

The authors provided the available original blot data to support the nCD100, CD72, and CD45 data reported in Figs 5, 7 and 9, but the data provided did not resolve the concerns and did not include matching data for all results in question. Corresponding author TL indicated that other underlying data for the reported experiments are unavailable due to issues including the amount of time that has passed (seven years) since the article’s publication, as well as the death (ALT), retirement (AT, AM, MC), and inability to contact (FP, LL, FM) some of the article’s authors.

In regards to the β-actin duplications, the authors commented that the same samples were analyzed in Figs 5, 7 and 9 and so the same β-actin controls were used for the corresponding panels of these figures. However, the β-actin data were not provided and we were unable to verify the authors’ claim based on the available data.

The authors disagreed with similarities of β-actin data across panels within figures (points 11–14, above). In regards to points 7–10 the authors raised that there are some differences between the data for which similarity concerns were raised. However, per our editorial assessment, the pixel similarities in the areas mentioned in points 7–10 are more similar than would be expected for different experimental results.

Overall the comments and data provided by the authors did not resolve the issues outlined above. The *PLOS ONE* Editors retract this article due to concerns about the validity and reliability of the reported results. We regret that the above issues were not identified and addressed during the article’s pre-publication peer review.

TL did not agree with the retraction and stands by the reported results. ML and DM disagree with the retraction. AT, FP, FM, LLS, MM, PC, AM and MC did not respond or could not be reached. ALT is deceased.
